# Epigenetic dichotomy in florid vs. gliotic proliferative diabetic retinopathy: hypomethylation of EGLN1 and MMP9 drives divergent pathogenic pathways in angiogenesis and fibrosis

**DOI:** 10.3389/fendo.2026.1664104

**Published:** 2026-03-25

**Authors:** Xiaotong Ren, Lijin Cui, Yao Yao, Yuzhe Qiu, Chenyue Yu, Jian Guo

**Affiliations:** 1 Department of Ophthalmology, The First Affiliated Hospital of Fujian Medical University, Fuzhou, Fujian, China; 2 Department of Ophthalmology, Fujian Provincial Geriatric Hospital, Fuzhou, Fujian, China

**Keywords:** EGLN1, florid PDR, gliotic PDR, hypomethylation, proliferative diabetic retinopathy (PDR)

## Abstract

**Objective:**

This exploratory, hypothesis-generating study aimed to investigate methylation differences in 16 angiogenesis- and fibrosis-related genes across diabetic retinopathy (DR) stages and proliferative diabetic retinopathy (PDR) subtypes (Florid and Gliotic), and to identify potential epigenetic biomarkers for disease progression.

**Methods:**

DNA methylation levels were analyzed in 38 diabetic patients stratified into three groups: Florid PDR (n=16), Gliotic PDR (n=13), and controls (no DR/non-proliferative DR, n=9). Targeted genes included *MMP9, EPO, AKR1B1, EGLN1, HIF1A, ICAM1, KDR, UCP1, SOD2, SERPINF1, PGF, RXRG, TGFB1, FLT1, FGF2*, and *VEGFA*. Methylation profiling was performed via MethylTarget sequencing.

**Results:**

In the Florid PDR group, *AKR1B1* (P = 0.039) and *MMP9* (P = 0.023) exhibited hypomethylation compared to controls. The Gliotic PDR group showed hypomethylation of *EPO* (P = 0.025), *KDR* (P = 0.023), *MMP9* (P = 0.014), and *UCP1* (P = 0.048) compared to controls. At the whole-promoter level, none of the 16 target genes showed statistically significant methylation differences between the Florid and Gliotic PDR groups. However, exploratory analysis of gene segments and individual CpG sites revealed nominal differences (e.g., in *EGLN1*) that warrant further investigation. Notably, *EGLN1* displayed hypomethylation at three CpG sites and two gene segments in Gliotic PDR (P<0.05).

**Conclusions:**

Distinct hypomethylation profiles in *AKR1B1, MMP9, EPO, KDR*, and *UCP1* were associated with PDR progression compared to no DR/non-proliferative controls. While no statistically significant differences were observed at the whole-promoter level for the 16 target genes between Florid and Gliotic PDR subtypes, segmental and CpG-level variations (particularly in *EGLN1*) suggest potential epigenetic heterogeneity. These preliminary findings highlight the need for further validation to assess their role as biomarkers or therapeutic targets.

## 1 Introduction

Diabetic retinopathy (DR), affecting approximately 22.27% of diabetics globally, is a leading cause of vision loss (1, 2). Proliferative diabetic retinopathy (PDR), the advanced stage (3), is a major public health problem in China, but epidemiological data on PDR in the Chinese population are still rather inconsistent (4–7). A 2023 bibliometric analysis showed that the global prevalence of PDR is 7.0%, and China is an important contributor to the research on DR (8). PDR is classified into two subtypes: Florid PDR, characterized by retinal neovascularization and vitreous hemorrhage (VH), and Gliotic PDR, marked by fibrovascular membranes (FVMs) formation (9). Gliotic PDR often portends a poorer prognosis than Florid PDR (10). Despite clinical distinctions, the molecular mechanisms driving these subtypes remain unclear, underscoring the need for biomarkers to predict progression and therapeutic targets.

DNA methylation plays a crucial role in regulating gene expression and has been implicated in various diseases, including DR (11). Epigenetic modifications in peripheral blood have also been explored as potential minimally invasive biomarkers of DR, highlighting the potential of epigenetic markers for DR diagnosis and monitoring (12–14). Furthermore, some aberrant methylation in genes were identified by some researches, suggesting potential therapeutic targets for PDR (15, 16). A latest finding suggests significant associations between the severity of DR and the DNA methylation levels of the genes *PSMA6* (proteasome 20S subunit alpha 6), *PSMB5* (proteasome 20S subunit beta 5), and *HIF1A* (hypoxia-inducible factor 1-alpha) (17). Another latest study found that miR-9–3 hypermethylation is associated with stages of diabetic retinopathy, with implications for serum levels of Vascular Endothelial Growth Factor (*VEGF*) (18).

These findings underscore the significance of investigating epigenetic modifications in understanding the DR pathogenesis. However, there is no published data on the methylation status of genes associated with different PDR types. But DNA methylation alterations have been observed in VH patients with other retinal diseases, including changes in *FZD4* exon 1 methylation (19). DNA methylation may have implications for the development of fibromembranous proliferation in the retina, which has been studied by FVMs from PDR patients (15). While the exact mechanisms linking DNA methylation to fibromembranous proliferation in diabetic retinopathy remain unclear, it is evident that understanding these processes is crucial for developing effective treatment strategies. Overall, the interplay among DNA methylation, vitreous hemorrhage, and fibromembranous proliferation in diabetic retinopathy is a complex and multifaceted topic that warrants further investigation. By elucidating the molecular mechanisms underlying these processes, researchers can identify novel therapeutic targets to manage this sight-threatening complication of diabetes.

However, methylation differences between PDR subtypes remain unexplored. To our knowledge, this is the first study to systematically analyze and compare promoter methylation patterns between the Florid and Gliotic subtypes of PDR. The 16 target genes (*AKR1B1, MMP9, EGLN1*, etc.) were selected in our study based on their established or strongly implicated roles in key DR-related pathways, including angiogenesis, hypoxia response, extracellular matrix remodeling/fibrosis, inflammation, and oxidative stress, as documented in prior literature. This study analyzed the promoter methylation status of these 16 genes in the subjects’ serum, identified specific methylation sites, and sought to identify biomarkers that could predict the trend of severe DR and the different types of PDR.

## 2 Methods

### 2.1 Ethical considerations

The study adhered to the Declaration of Helsinki and was approved by the Ethics Committee of the First Affiliated Hospital of Fujian Medical University. Each patient has provided written informed consent prior to enrollment in the study. Thirty-eight diabetic patients (23 female) were enrolled.

### 2.2 Participants

Diabetic patients (with Type 2 Diabetes Mellitus, T2DM) who requested fundus examination at First Affiliated Hospital of Fujian Medical University Eye Center from November 2017 to September 2018 were enrolled. Diabetes was diagnosed according to the diagnostic criteria by the American Diabetes Association (ADA) (20). Patients were excluded from the study if they had other diseases that cause retinal hemorrhage and proliferation, such as retinal vein occlusion, retinal detachment, or macular degeneration. In addition, patients who suffered severe or complex systemic disease were also excluded. Patients who were incapable of carrying out study-related visits were also excluded. All patients were under standard glycemic control regimens, including oral hypoglycemic agents and/or insulin. None of the included patients had received prior anti-VEGF therapy, intravitreal steroids, or pan-retinal photocoagulation (PRP) for PDR.

### 2.3 DR classification

DR grading was based on the fundus examination carried out by the same two senior ophthalmologists. Florid PDR was defined by the presence of active retinal neovascularization with or without vitreous hemorrhage, while Gliotic PDR was characterized by predominant fibrovascular membrane formation with signs of gliosis and traction (9).

Patients were stratified into three groups: Florid PDR (n=16): Neovascularization-dominant; Gliotic PDR (n=13): Fibrovascular membrane-dominant; Control group (n=9), termed “Non-Proliferative DR (NPDR/NDR) group” for clarity, consisted of patients with No DR (NDR, n=4) or non-proliferative DR (NPDR, n=5). NDR patients were enrolled during routine annual diabetic eye screening. Including NPDR allowed us to compare methylation profiles across the continuum of DR severity, from non-proliferative to proliferative stages.

### 2.4 Biochemical parameters

All patients in this study were subjected to a detailed medical history taking, including age, gender, height, weight, Body Mass Index (BMI), history of diabetes and hypertension, use of diabetes medication and hypertension medication, history of smoking and drinking, daily exercise, family history, fasting blood glucose level, and glycated hemoglobin (HbA1c).

### 2.5 Sampling of blood for DNA extraction and serum preparation

Peripheral venous blood was collected for serum preparation. The samples were incubated undisturbed for 30 minutes at room temperature and then centrifuged. The serum was transferred into fresh 2 mL tubes, frozen, and stored at −80°C until analysis. DNA isolation from frozen whole-blood samples stored at −80 °C using the phenol–chloroform extraction method was performed in the biobank setting.

### 2.6 Targeted DNA methylation assessment

The DNA methylation levels of specific CpG sites were determined by MethylTarget sequencing (Genesky Biotechnologies Inc., Shanghai, China), a method using next-generation sequencing-based multiple targeted CpG methylation analysis (21, 22). Primer design and validation were performed by Methylation Primer software on bisulfate-converted DNA. Primer sets were designed to flank each targeted CpG site in 100–300 nucleotide regions. Genomic DNA was extracted from frozen samples using Genomic Tip-500 columns (Qiagen, Valencia, CA, USA), and bisulfite-converted DNA was extracted using the EZ DNA Methylation™-GOLD Kit (Zymo Research, CA, USA) according to the manufacturer’s protocols. After PCR amplification (HotStarTaq polymerase kit, TAKARA, Tokyo, Japan) and library construction, samples were sequenced (Illumina HiSeq Benchtop Sequencer, CA, USA) using the paired-end sequencing protocol according to the manufacturer’s guidelines (23).

Gene segments and CpG sites were numbered sequentially according to their genomic positions relative to the transcription start site. The format “*Gene_X_Y_Z*” indicates: X = amplicon number, Y = segment identifier within the amplicon, and Z = nucleotide position relative to the amplicon start. For example, “*EGLN1_1_2_73*” refers to amplicon 1, segment 2, CpG site at position 73.

### 2.7 Power analysis statement

Sample size was determined based on previous epigenetic studies in DR, with an estimated effect size of 0.8, alpha of 0.05, and power of 80%.

### 2.8 Statistical analysis

SPSS software version 23 (SPSS Inc, Chicago, IL, USA) was used for statistical analysis. Data normality was assessed using the Kolmogorov-Smirnov test. The descriptive data were presented as means and standard deviations (SD). Methylation levels are expressed as median (%)and extreme values (%). Binary logistic regression was performed to assess associations between methylation levels and PDR subtypes, with results expressed as odds ratios (OR) and 95% confidence intervals (CI). Statistics were performed by t-test and ANOVA. Student’s t-test was used for comparisons between two groups. One-way ANOVA was used to compare three or more groups. P-value of less than 0.05 was considered statistically significant.

## 3 Results

### 3.1 Characteristics of patients in each group

The study recruited 38 patients, including 23 women. The basic characteristics of patients in each group are shown in **Table 1**. Groups were not fully matched for age and gender; however, these variables were adjusted for in regression analyses.

**Table 1:** Characteristics of patients in each group.

Index	Florid PDR group (n=16)	Gliotic PDR group (n=13)	Control group (n=9 )
Sex (male, female)	6/10	7/6	2/7
Age (Years)	50.8 ± 4.5	55.5 ± 9.5	64.5 ± 4.5
History of diabetes (Years)	12.4 ± 2.1	11.3 ± 1.4	5.0 ± 1.9
Hypertension (Number)	10(62.5%)	7(53.8%)	3(33.3%)
BMI(kg/m^2^)	24.87 ± 2.43	22.86 ± 0.98	25.28 ± 4.45
Fasting blood glucose level(mmol/L)	8.5 ± 1.0	10.3 ± 1.2	7.8 ± 0.3
HbA1c (%)	7.7 ± 0.9	8.4 ± 0.5	6.0 ± 0.3

Data are presented as mean ± SD for continuous variables and as n (%) or n (male/female) for categorical variables. Groups were not fully matched for age and sex. BMI, Body mass index.

### 3.2 DNA methylation levels of promoter parts of genes between PDR and controls

**Figure 1:**
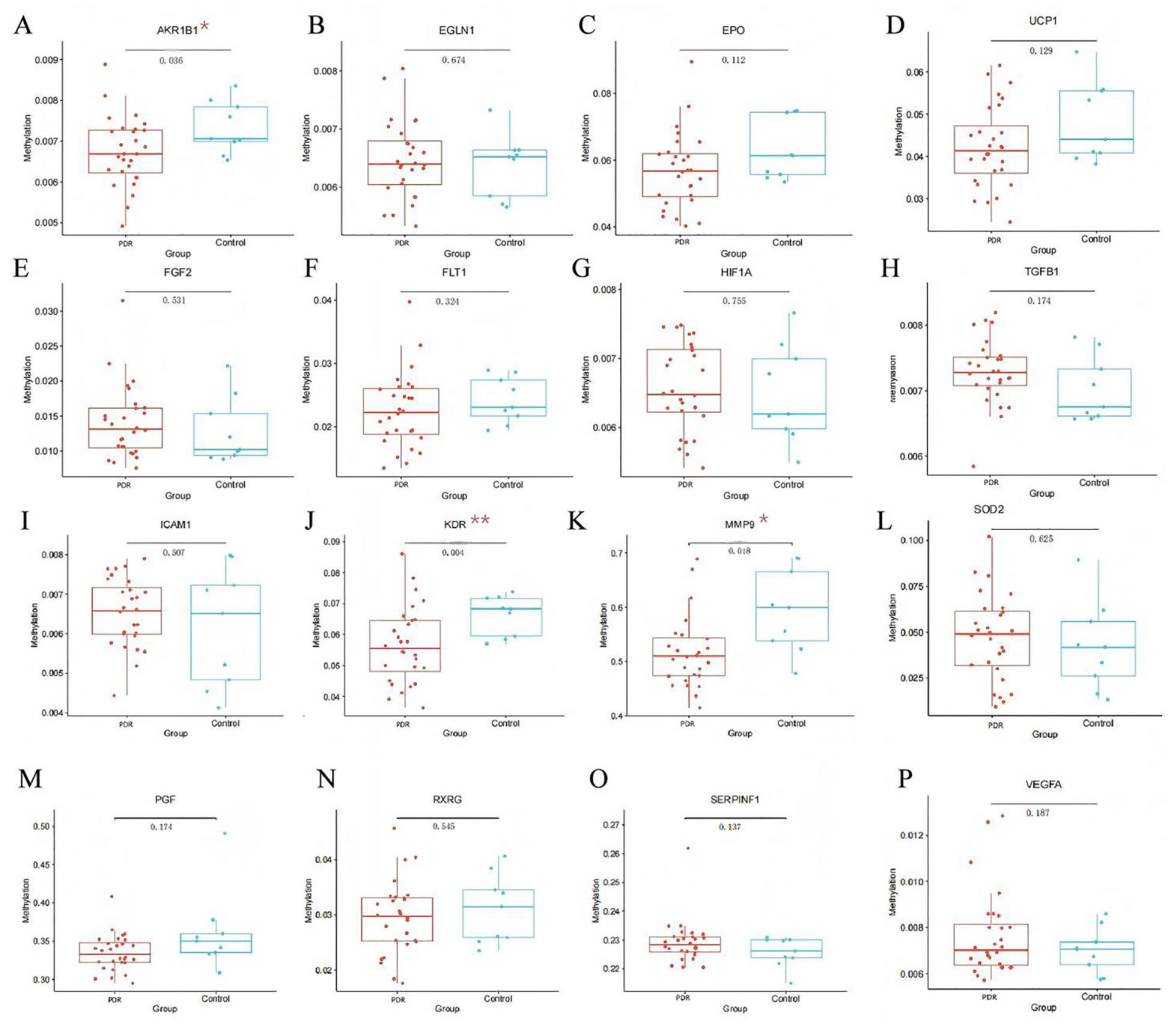
DNA methylation levels of promoter regions of selected genes in PDR (Florid + Gliotic) versus the Control group. *MMP9*, Matrix Metallopeptidase 9; *EPO*, Erythropoietin; *AKR1B1*: Aldo-Keto Reductase Family 1 Member B1; *EGLN1*, Egl-9 Family Hypoxia Inducible Factor 1; *HIF1A*, Hypoxia Inducible Factor 1 Subunit Alpha; *ICAM1*, Intercellular Adhesion Molecule 1; *KDR*, Kinase Insert Domain Receptor; *UCP1*, Uncoupling Protein 1; *SOD2*, Superoxide Dismutase 2; *SERPINF1*, Serpin Family F Member 1; *PGF*, Placental Growth Factor; *RXRG*, Retinoid X Receptor Gamma; *TGFB1*, Transforming Growth Factor Beta 1; *FLT1*, Fms Related Receptor Tyrosine Kinase 1; *FGF2*, Fibroblast Growth Factor 2; *VEGFA*, Vascular Endothelial Growth Factor A. Box plots with superimposed dots show methylation levels (%) of each gene promoter. Each dot represents an individual sample. The PDR group (n=29) includes Florid (n=16) and Gliotic (n=13) subtypes; the Control group (n=9) includes NPDR (n=5) and NDR (n=4) patients. Center line, median; box, interquartile range (IQR); whiskers, 1.5× IQR. P-values were calculated using Student’s t-test. All P-values are uncorrected for multiple comparisons and should be interpreted as exploratory. *P < 0.05, **P < 0.01. All gene symbols are italicized.

As shown in **Figure 1**, *AKR1B1* (P = 0.036), *KDR* (P = 0.004), and *MMP9* (P = 0.018) showed low methylation in PDR patients compared to controls.

### 3.3 DNA methylation levels of promoter parts of genes among different DR severity stages and types

**Figure 2:**
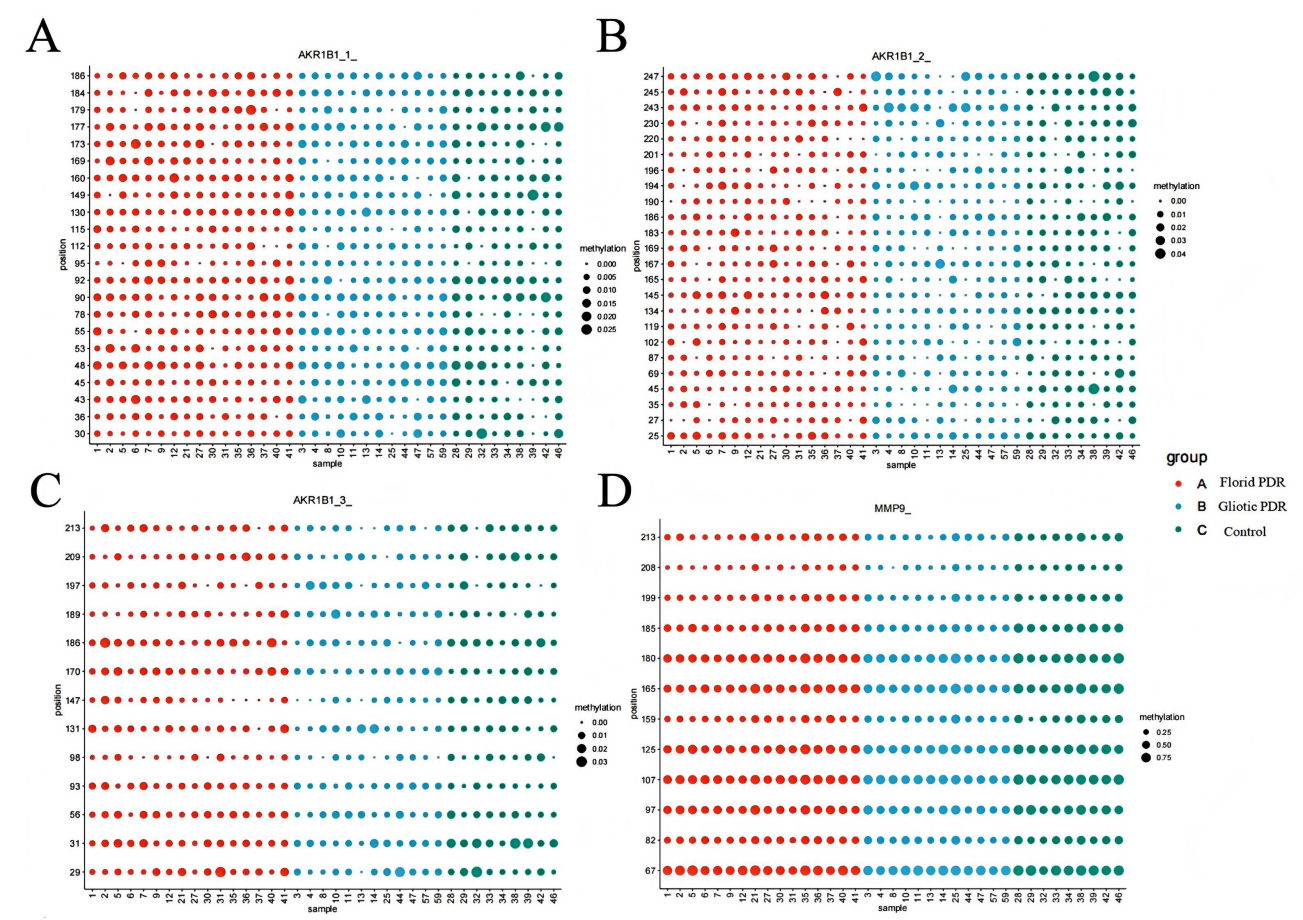
DNA methylation levels of *AKR1B1* and *MMP9* in Florid PDR, Gliotic PDR, and Control groups. *MMP9*, Matrix Metallopeptidase 9; *AKR1B1*, Aldo-Keto Reductase Family 1 Member B1, Bubble plots show methylation levels (%) of *AKR1B1* and *MMP9*. The x−axis represents individual sample IDs; the y−axis indicates specific gene loci. Bubble size is proportional to the methylation level (%). Colors represent the three study groups: red, Florid PDR (n=16); blue, Gliotic PDR (n=13); green, Control (NPDR/NDR, n=9). P−values were calculated using one−way ANOVA with Tukey’s post−hoc test. All P−values are uncorrected for multiple comparisons and should be interpreted as exploratory. All gene symbols are italicized.

As shown in **Figure 2**; **Table 2**, *AKR1B1* (P = 0.039) and *MMP9* (P = 0.023) showed low methylation in the Florid PDR group compared to the control group.

**Table 2:** Genes showing nominally significant methylation differences between florid PDR and control groups.

Gene	Florid PDR group (n=16) Median (range)	Control group (n=9) Median (range)	P
*AKR1B1*, %	0.67 (0.57-0.76)	0.71 (0.65-0.84)	0.039
*MMP9*, %	50.7 (43.7-68.9)	60.0 (47.8-69.1)	0.023

Data are presented as median (minimum-maximum). P-values were calculated using Student’s t-test. Only genes with nominally significant differences (P < 0.05) are shown; genes without significant differences are not listed. All P-values are uncorrected for multiple comparisons and should be interpreted as exploratory and hypothesis-generating. *MMP9*, Matrix Metallopeptidase 9; *AKR1B1*: Aldo-Keto Reductase Family 1 Member B1. All gene symbols are italicized.

**Figure 3:**
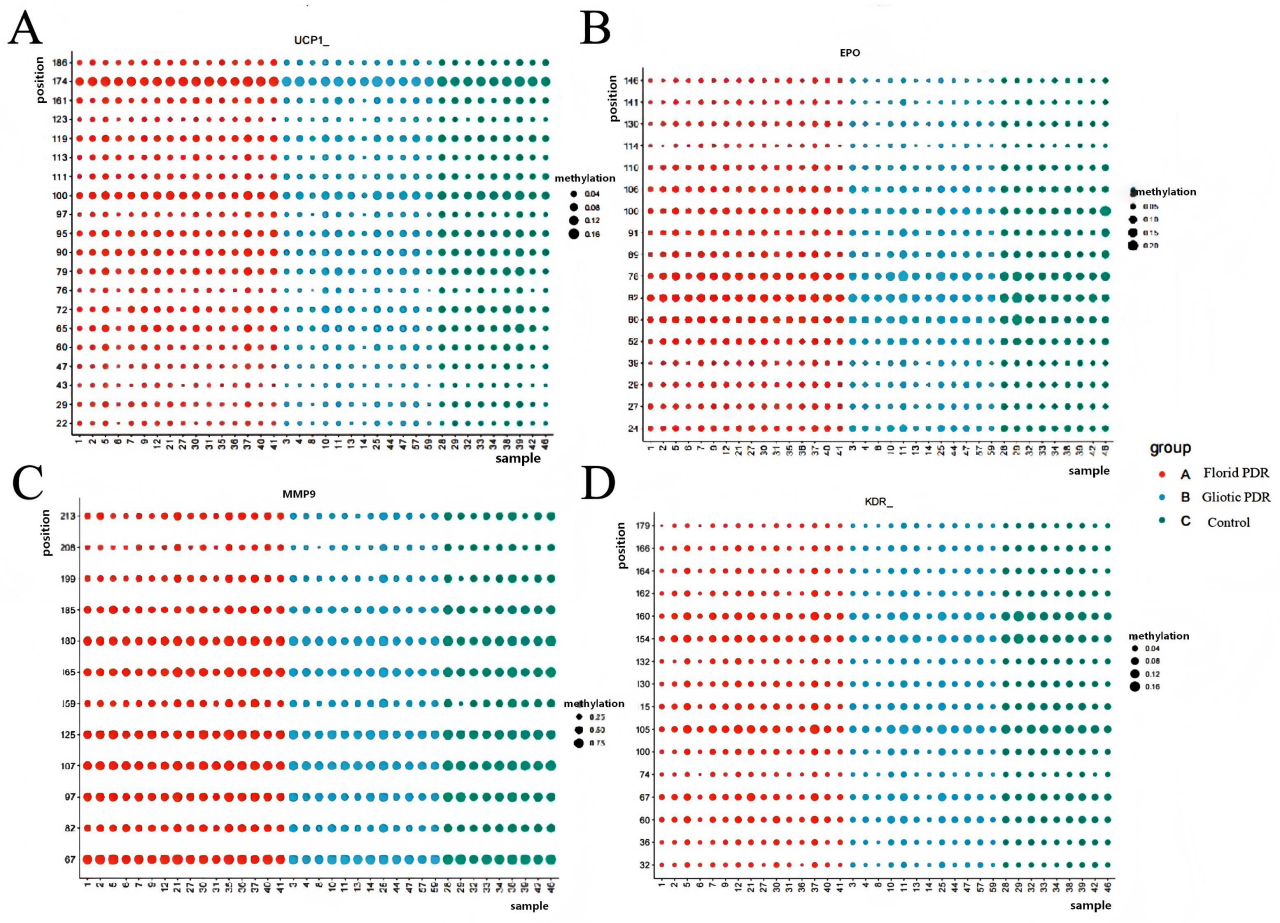
DNA methylation levels of *UCP1*, *EPO*, *KDR* and *MMP9* in Florid PDR, Gliotic PDR, and Control groups. *MMP9*, Matrix Metallopeptidase 9; *EPO*, Erythropoietin; *KDR*, Kinase Insert Domain Receptor; *UCP1*, Uncoupling Protein 1. Bubble plots show methylation levels (%) of *UCP1*, *EPO*, *KDR*, and *MMP9*. The x−axis represents individual sample IDs; the y−axis indicates specific gene loci. Bubble size is proportional to the methylation level (%). Colors represent the two study groups: red, Florid PDR (n=16); blue, Gliotic PDR (n=13); green, Control (NPDR/NDR, n=9). P−values were calculated using one−way ANOVA with Tukey’s post−hoc test. All P−values are uncorrected for multiple comparisons and should be interpreted as exploratory. All gene symbols are italicized.

In **Figure 3**; **Table 3**, the *EPO* (P = 0.025), KDR (P = 0.023), *MMP9* (P = 0.014), and *UCP1* (P = 0.048) genes in the Gliotic group are hypomethylated compared to the control group.

**Table 3:** Genes showing nominally significant methylation differences between gliotic PDR and the control groups.

Gene	Gliotic PDR group (n=13) Median (range)	Control group (n=9) Median (range)	P
*EPO*, %	5.6 (4.0-7.0)	6.1 (5.3-7.5)	0.025
*KDR*, %	5.5 (3.9-8.6)	6.8 (5.7-7.4)	0.023
*MMP9*, %	51.4 (41.5-67.0)	60.0 (47.8-69.1)	0.014
*UCP1*, %	4.1 (2.9-5.9)	4.4 (3.8-6.5)	0.048

Data are presented as median (minimum-maximum). P-values were calculated using Student’s t-test. Only genes with nominally significant differences (P < 0.05) are shown; genes without significant differences are not listed. All P-values are uncorrected for multiple comparisons and should be interpreted as exploratory and hypothesis-generating. *MMP9*, Matrix Metallopeptidase 9; *EPO*, Erythropoietin; *KDR*, Kinase Insert Domain Receptor; *UCP1*, Uncoupling Protein 1. All gene symbols are italicized.

For all 16 target genes, no statistically significant differences in whole-promoter methylation levels were detected between the Florid and Gliotic PDR groups (P > 0.05 for all genes).

### 3.4 Methylation level difference of gene segments and CpG sites between gliotic and florid PDR group

Although no statistically significant differences were detected at the whole-promoter level for the 16 target genes between the two PDR subtypes, exploratory analysis of finer-resolution regions revealed several nominally significant differences at the gene segment and individual CpG site levels.

**Figure 4:**
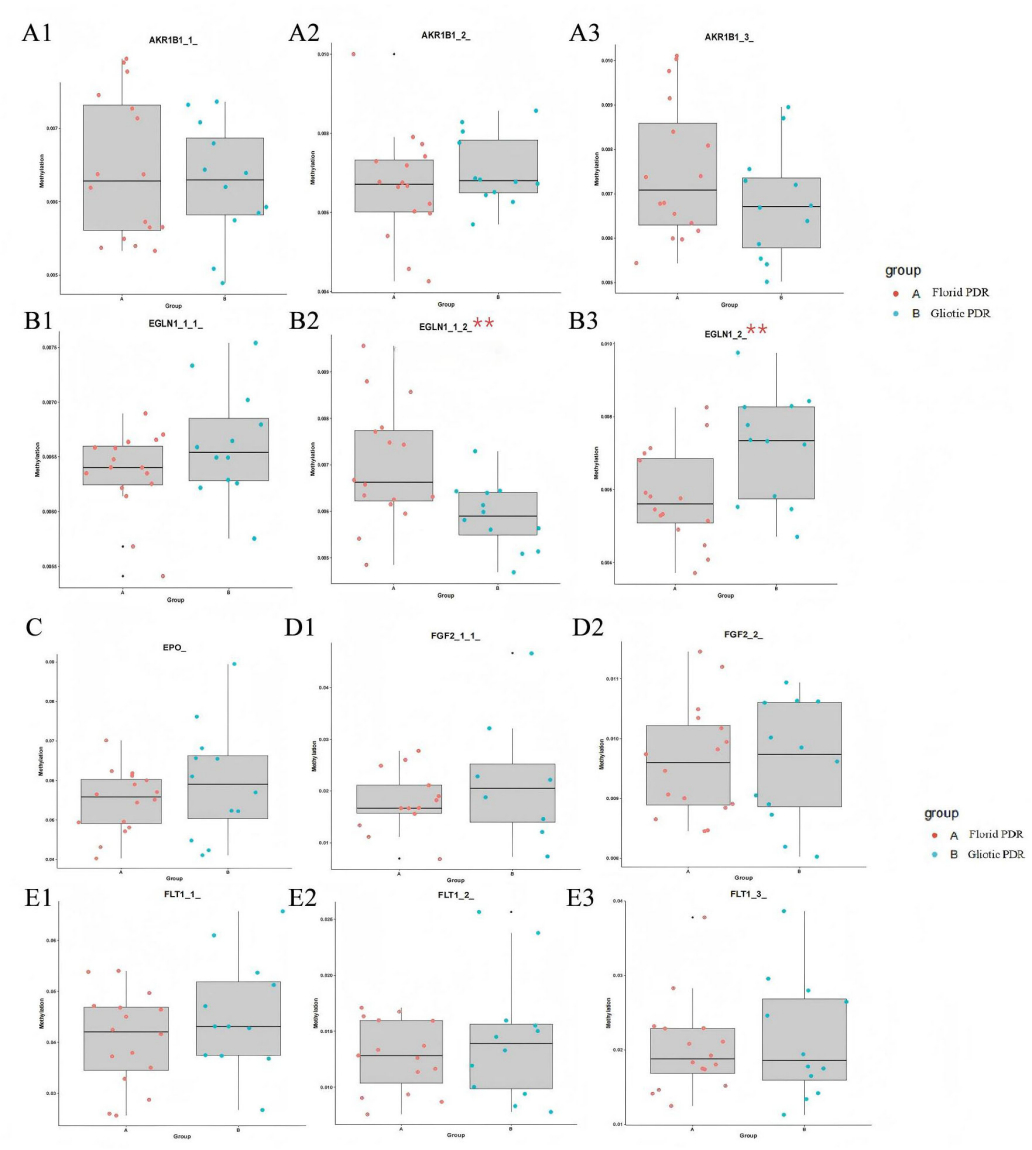
Methylation level difference of *AKR1B1*, *EGLN1*, *EPO*, *FGF2*, and *FLT1* gene segments between Gliotic and Florid PDR group. *EPO*, Erythropoietin; *AKR1B1*, Aldo-Keto Reductase Family 1 Member B1; *EGLN1*, Egl-9 Family Hypoxia Inducible Factor 1; *FLT1*, Fms Related Receptor Tyrosine Kinase 1; *FGF2*, Fibroblast Growth Factor 2. Box plots with superimposed dots compare methylation levels (%) at specific gene segments. Each dot represents an individual sample. Gliotic PDR (n=13) and Florid PDR (n=16) are shown in blue and red, respectively. Center line, median; box, interquartile range (IQR); whiskers, 1.5× IQR. P-values were calculated using Student’s t-test. All P-values are uncorrected for multiple comparisons; these comparisons are exploratory and hypothesis-generating. *P < 0.05, **P < 0.01. All gene symbols are italicized.

**Figure 5:**
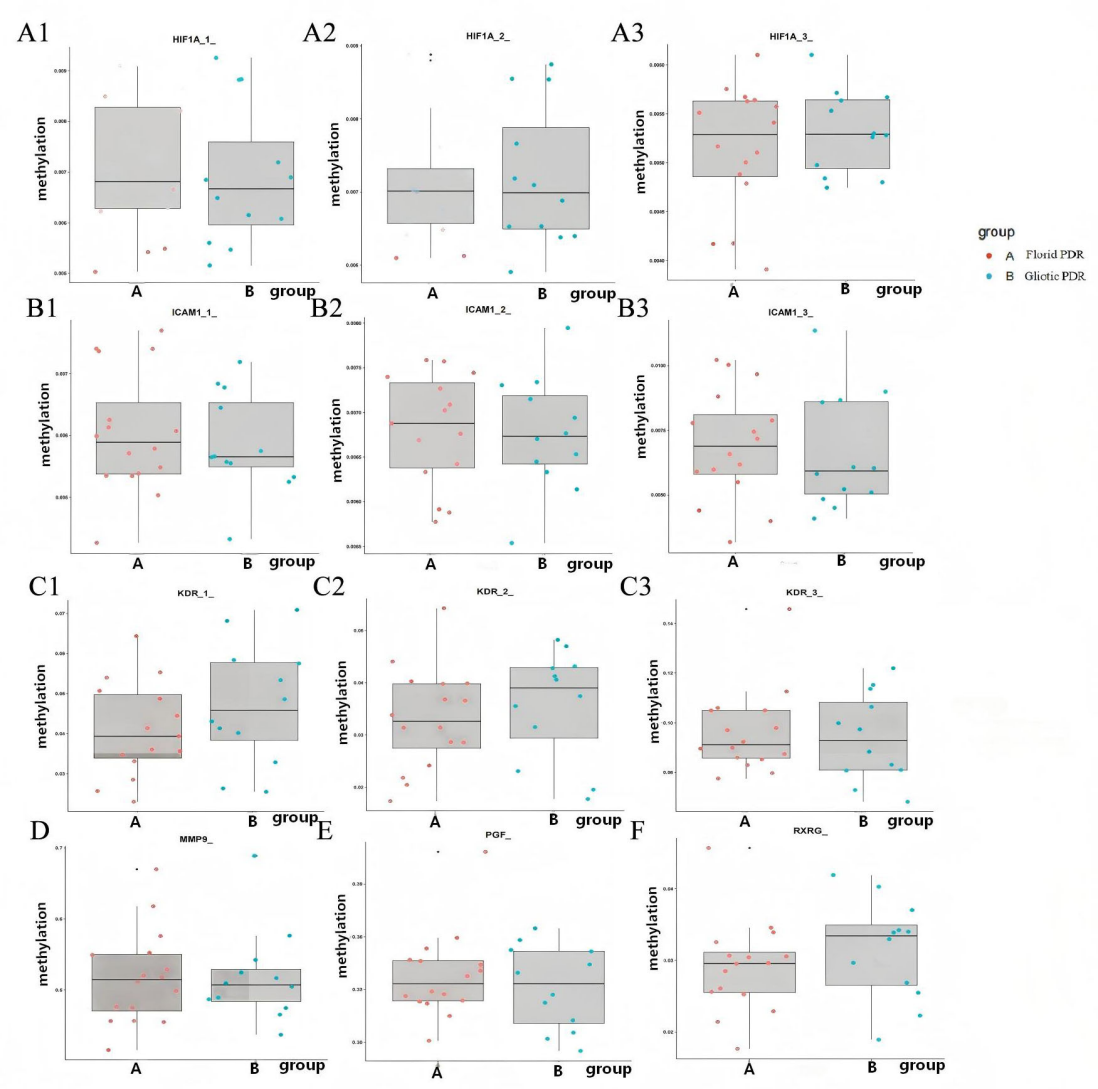
Methylation level difference of *HIF1A*, *ICAM1*, *KDR*, *MMP9*, *PGF*, and *RXRG* gene segments between Gliotic and Florid PDR group. *MMP9*, Matrix Metallopeptidase 9; *HIF1A*, Hypoxia Inducible Factor 1 Subunit Alpha; *ICAM1*, Intercellular Adhesion Molecule 1; *KDR*, Kinase Insert Domain Receptor; *PGF*, Placental Growth Factor; *RXRG*, Retinoid X Receptor Gamma; *TGFB1*, Transforming Growth Factor Beta 1. Box plots with superimposed dots compare methylation levels (%) at specific gene segments. Each dot represents an individual sample. Gliotic PDR (n=13) and Florid PDR (n=16) are shown in blue and red, respectively. Center line, median; box, interquartile range (IQR); whiskers, 1.5× IQR. P-values were calculated using Student’s t-test. All P-values are uncorrected for multiple comparisons; these comparisons are exploratory and hypothesis-generating. All gene symbols are italicized.

**Figure 6:**
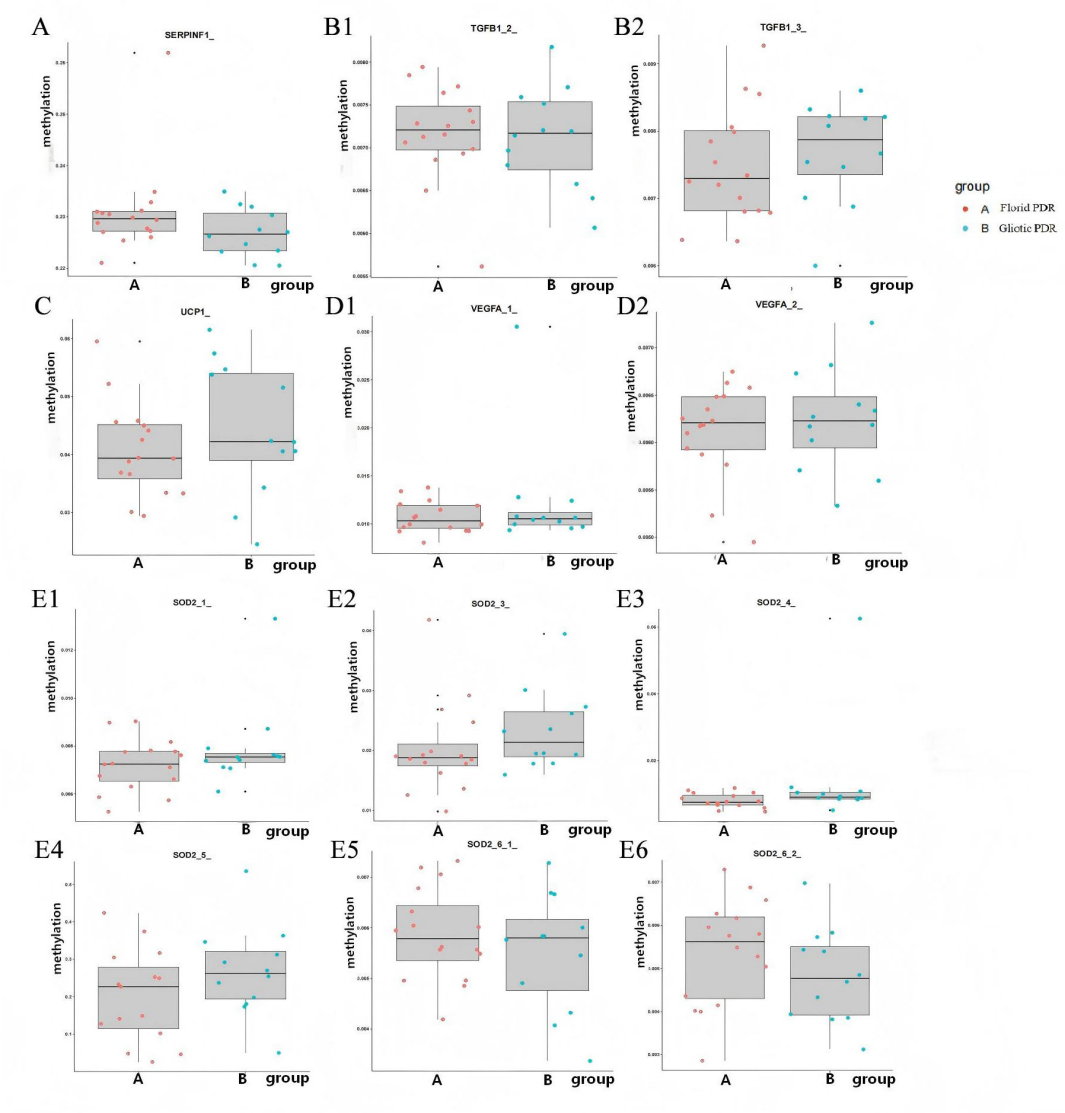
Methylation level difference of *SERPINF1*, *TGFB1, UCP1, VEGFA*, and *SOD2* gene segments between Gliotic and Florid PDR group. *UCP1*, Uncoupling Protein 1; *SOD2*, Superoxide Dismutase 2; *SERPINF1*, Serpin Family F Member 1; *TGFB1*, Transforming Growth Factor Beta 1; *VEGFA*, Vascular Endothelial Growth Factor A. Box plots with superimposed dots compare methylation levels (%) at specific gene segments. Each dot represents an individual sample. Gliotic PDR (n=13) and Florid PDR (n=16) are shown in blue and red, respectively. Center line, median; box, interquartile range (IQR); whiskers, 1.5× IQR. P-values were calculated using Student’s t-test. All P-values are uncorrected for multiple comparisons; these comparisons are exploratory and hypothesis-generating. All gene symbols are italicized.

**Figures 4**–**6** show box plots with superimposed dots comparing methylation levels of all analyzed gene segments between the Gliotic and Florid PDR groups. As shown in these figures, most gene segments exhibited comparable methylation levels across the two subtypes, whereas some segments showed a trend toward differential methylation.

**Table 4** lists the gene segments that reached nominal statistical significance (P < 0.05, uncorrected) in the comparison between Gliotic and Florid PDR groups. Notably, two segments of the EGLN1 gene (*EGLN1_1_2_* and *EGLN1_2_*) showed significantly lower methylation in the Gliotic PDR group compared to the Florid PDR group.

**Table 4:** Gene segments showing nominal methylation differences between gliotic and florid PDR groups.

Gene segment*	Gliotic PDR group (n=13) Median (range)	Florid PDR group (n=16) Median (range)	P
*EGLN1_1_2_*	0.7 (0.5-1.0)	0.6 (0.5-0.7)	0.015
*EGLN1_2_*	0.7 (0.5-1.1)	0.5 (0.4-0.8)	0.015

Data are presented as median (minimum-maximum) methylation level (%). *Gene segment nomenclature is defined in Section 2.6 (Methods). P-values were calculated using Student’s t-test. Only segments with nominally significant differences (P < 0.05) are shown; those without significant differences are not listed. All P-values are uncorrected for multiple comparisons; these comparisons are exploratory and hypothesis-generating. All gene symbols are italicized. *EGLN1*, Egl-9 Family Hypoxia Inducible Factor 1. All gene symbols are italicized.

**Table 5** presents the individual CpG sites that exhibited nominal statistical significance (P < 0.05, uncorrected) between the two subtypes. A total of 20 CpG sites across multiple genes showed differential methylation, including sites within *EGLN1, SOD2, AKR1B1, FLT1, HIF1A, PGF, SERPINF1*, and *TGFB1*.

**Table 5:** Individual CpG sites showing nominal methylation differences between gliotic and florid PDR groups.

Gene segment*	Site†	Gliotic PDR group (n=13) Median (range)	Florid PDR group (n=16) Median (range)	P
*AKR1B1_1_*	115	0.6 (0.1-1.1)	0.3 (0-0.7)	0.042
*AKR1B1_2_*	145	0.7 (0.4-2.2)	0.5 (0-1.1)	0.035
*AKR1B1_2_*	194	0.3 (0-2.1)	0.9 (0.2-2.8)	0.039
*EGLN1_1_1_*	78	0.5 (0.2-0.7)	0.7 (0.4-0.8)	0.007
*EGLN1_1_1_*	150	0.8 (0.6-1.2)	1.0 (0.6-1.3)	0.010
*EGLN1_1_2_*	73	0.6 (0-2.2)	0.2 (0-0.7)	0.007
*EGLN1_2_*	85	0.5 (0.3-1.5)	0.2 (0-1.1)	0.047
*FLT1_2_*	111	1.1 (0.5-1.5)	1.5 (0.9-3.5)	0.017
*HIF1A_1_*	189	1.1 (0-2.4)	0.7 (0-1.5)	0.014
*PGF_*	187	39.2(34.3-48.0)	36.6 (29.7-44.9)	0.020
*SERPINF1_*	197	32.1(29.7-33.9)	30.9 (29.0-32.4)	0.011
*SOD2_1_*	40	0.5 (0-1.1)	1.2 (0.8-2.2)	0.000
*SOD2_1_*	76	1.2 (0-2.2)	0.8 (0.5-1.2)	0.005
*SOD2_1_*	146	1.3 (0.5-2.1)	0.8 (0.2-3.7)	0.029
*SOD2_4_*	39	0.7 (0-2.7)	1.3 (0-3.5)	0.031
*SOD2_4_*	100	0.4 (0-1.5)	1 (0-3.4)	0.020
*TGFB1_2_*	87	0.8 (0.3-1.3)	0.5 (0.2-1)	0.023

Data are presented as median (minimum-maximum) methylation level (%). *Gene segment nomenclature is defined in Section 2.6 (Methods). †CpG sites are numbered according to their position within each amplicon (see Section 2.6). P-values were calculated using Student’s t-test. Only sites with nominally significant differences (P < 0.05) are shown; those without significant differences are not listed. All P-values are uncorrected for multiple comparisons; these comparisons are exploratory and hypothesis-generating. *AKR1B1*, Aldo-Keto Reductase Family 1 Member B1; *EGLN1*, Egl-9 Family Hypoxia Inducible Factor 1; *HIF1A*, Hypoxia Inducible Factor 1 Subunit Alpha; *SOD2*, Superoxide Dismutase 2; *SERPINF1*, Serpin Family F Member 1; *PGF*, Placental Growth Factor; *FLT1*, Fms Related Receptor Tyrosine Kinase 1; *TGFB1*, Transforming Growth Factor Beta 1. All gene symbols are italicized.

Notably, within the *EGLN1* gene, locus-specific patterns were observed. In the *EGLN1_1_2_* and *EGLN1_2_* segments, which showed overall segment-level hypomethylation in Florid PDR (**Table 4**), the constituent CpG sites (sites 73 and 85, respectively) also demonstrated significantly lower methylation in the Florid group, consistent with the segment-level findings. In contrast, within the *EGLN1_1_1_* segment—which did not reach statistical significance at the whole-segment level—two CpG sites (sites 78 and 150) showed significantly higher methylation in the Florid PDR group, suggesting that segment-level non-significance may reflect averaging across sites with opposing or heterogeneous methylation patterns. For other genes, multiple CpG sites showed differential methylation between the two subtypes, with varying directions of effect.

Given the exploratory nature of these analyses and the lack of multiple testing correction, all findings presented in **Table 4** and **Table 5** should be interpreted as hypothesis-generating signals requiring validation in independent cohorts.

## 4 Discussion

DNA methylation, a key epigenetic regulator, is implicated in DR pathogenesis (5–7). Prior studies identified aberrant methylation in genes such as *HIF1A* and *PSMA6* in DR severity (10, 11). However, methylation differences between PDR subtypes remain unexplored. This study analyzed promoter methylation of 16 genes linked to angiogenesis and fibrosis in Florid and Gliotic PDR to examine the effect of methylation difference on different stages and types of diabetic retinopathy. It is important to frame this as an exploratory, hypothesis-generating investigation. The following discussion interprets the observed methylation differences to generate hypotheses for future research rather than to establish definitive pathogenic mechanisms.4.1 Hypomethylation patterns associated with PDR progression.

In the present study, the Florid PDR group exhibited significantly lower *AKR1B1* promoter methylation compared to the control group (**Table 2**). Previous studies have implicated *AKR1B1* in the pathogenesis of diabetic retinopathy (24–26). *AKR1B1* encodes aldose reductase, the first and rate-limiting enzyme of the polyol pathway, which reduces glucose to sorbitol and is expressed in retinal capillary pericytes (27), with elevated expression observed in the retinas of hyperglycemia-induced lesions (28, 29). The hypomethylation observed in our study is consistent with findings in diabetic nephropathy, where *AKR1B1* hypomethylation was associated with albuminuria and proposed to contribute to extracellular matrix degradation and polyol pathway activation (30). Experimental evidence from animal models further supported this link: *AKR1B1* knockdown in rat lenses significantly reduced aldose reductase activity and sorbitol accumulation under hyperglycemic conditions (31). Taken together, these observations suggest that *AKR1B1* hypomethylation in Florid PDR may enhance polyol pathway flux, contributing to oxidative stress and the angiogenic phenotype characteristic of this subtype. However, given the modest absolute difference in methylation (0.67% vs. 0.71%) and the exploratory nature of this analysis, this finding should be interpreted with caution and requires functional validation.

In the Gliotic PDR group, four genes—*EPO*, *KDR*, *UCP1*, and *MMP9*—showed significant hypomethylation compared to controls (**Table 3**). Among these, *EPO*, *KDR*, and *UCP1* are discussed in this section, while *MMP9* is addressed separately below due to its unique hypomethylation pattern in both Florid and Gliotic PDR subtypes, which distinguishes it from the Gliotic-specific findings.

Erythropoietin (EPO) is a glycoprotein hormone that regulates erythropoiesis and has been implicated in retinal angiogenesis (32). Under normoxic conditions, the EPO promoter is typically methylated, suppressing transcription; hypoxic conditions lead to demethylation and increased expression (33, 34). Consistent with this regulatory mechanism, previous studies have shown that *EPO* hypomethylation correlates with increased *EPO* expression and promotes retinal angiogenesis in PDR (35–37). The hypomethylation observed in our Gliotic PDR group may therefore contribute to the fibrovascular proliferation characteristic of this subtype.

Vascular Endothelial Growth Factor Receptor (VEGFR-2)/KDR is the primary receptor mediating VEGF-induced angiogenesis (38). Hypomethylation of the *KDR* promoter has been shown to increase *KDR* protein synthesis and upregulate arachidonic acid signaling (39), which plays a regulatory role in endothelial cell proliferation and tubulogenesis (40). Our finding of *KDR* hypomethylation in Gliotic PDR suggests that this angiogenic pathway may be epigenetically primed even in the fibrosis-dominant phenotype, potentially contributing to the vascular component of fibrovascular membranes.

Uncoupling protein 1 (*UCP1*) is a mitochondrial inner membrane protein involved in energy metabolism and oxidative stress regulation (41). While *UCP1* has been primarily studied in the context of adipose tissue browning and metabolic disease (42), emerging evidence suggests it may also play a protective role in vascular health. *UCP1* deficiency has been shown to exacerbate endothelial dysfunction and vascular inflammation in mouse models (43). The modest hypomethylation of *UCP1* observed in our Gliotic PDR group (4.1% vs. 4.4%, P = 0.048) is paradoxical, as it would be expected to increase *UCP1* expression and potentially mitigate inflammation. This may reflect a compensatory anti-fibrotic response or context-dependent effects in the retinal microenvironment, but the small magnitude of the difference (0.3%) warrants caution in interpretation and requires validation in larger cohorts.

As noted above, *MMP9* showed significant hypomethylation both in the Florid PDR and Gliotic PDR, respectively compared to controls (**Tables 2**, **3**). This dual-subtype pattern distinguishes it from the Gliotic-specific findings discussed above and suggests a broader role in PDR pathogenesis (44–47). Previous studies have demonstrated that under high glucose conditions, the *MMP9* promoter undergoes active demethylation through interactions between *FOXO1* and RNA polymerase II, as well as recruitment of the demethylase Tet2 (45, 48, 49). This results in increased MMP-9 expression, which damages mitochondria and accelerates apoptosis of retinal capillary cells—events that precede clinically evident retinopathy (45). The consistent hypomethylation observed across both PDR subtypes in our study suggests that *MMP9* epigenetic dysregulation may represent a common pathway in the transition from non-proliferative to proliferative disease, independent of the subsequent divergence into angiogenic (Florid) versus fibrotic (Gliotic) phenotypes. This interpretation is supported by studies in other disease contexts: in systemic lupus erythematosus, *MMP9* promoter hypomethylation correlated with disease severity markers (50), and in cerebrovascular disease, *MMP9* hypomethylation was associated with transient ischemic attack and mild ischemic stroke (51). These parallels suggest that *MMP9* methylation status may serve as a generalizable marker of tissue remodeling and inflammation across different pathological conditions. In the context of PDR, *MMP9* hypomethylation may represent an early epigenetic event that creates a permissive environment for both neovascularization and fibrosis, with the ultimate clinical phenotype determined by additional subtype-specific epigenetic modifications such as those observed in Egl-9 Family Hypoxia Inducible Factor 1(*EGLN1*).

### 4.2 *EGLN1* exhibits complex, locus-specific methylation patterns between PDR subtypes

Although no statistically significant differences were observed at the whole-promoter level across the 16 target genes between the two PDR subtypes, exploratory analysis of gene segments and individual CpG sites revealed several nominally significant differences within the *EGLN1* gene, with notable locus-specific heterogeneity.

There were some consistent findings at the segment and CpG levels. The *EGLN1_1_2_* and *EGLN1_2_* segments showed significantly lower methylation in Florid PDR compared to Gliotic PDR at the whole-segment level (**Table 4**). This pattern was corroborated at the individual CpG level, with sites 73 (*EGLN1_1_2_*) and 85 (*EGLN1_2_*) also exhibiting hypomethylation in the Florid group (**Table 5**). This concordance suggests that the differential methylation in these regions is robust at multiple resolution levels and may have functional implications for *EGLN1* expression. In contrast, within the *EGLN1_1_1_* segment—which did not reach statistical significance at the whole-segment level—two CpG sites (sites 78 and 150) showed significantly higher methylation in the Florid PDR group. This apparent discrepancy highlights an important biological consideration: gene segments may contain CpG sites with opposing methylation patterns that cancel each other out when averaged across the entire segment. Such intra-segmental heterogeneity may reflect differential regulation of individual CpG sites by distinct transcription factors or chromatin modifiers, and has been increasingly recognized in epigenetic studies of complex diseases (11).

*EGLN1* (also known as PHD2) encodes a prolyl hydroxylase that serves as a key oxygen sensor, targeting hypoxia-inducible factor-1α(HIF-1α) for proteasomal degradation under normoxic conditions (52). Previous studies have shown that hypomethylation of *EGLN1* regulatory regions is associated with enhanced expression in response to hypoxic stress (53), representing a negative feedback mechanism that fine-tunes HIF-1α activity by promoting its degradation when oxygen levels normalize. In contrast, the predominantly higher methylation of *EGLN1* CpG sites observed in Gliotic PDR in our study would be expected to have the opposite effect: reduced *EGLN1* expression, leading to impaired HIF-1α degradation and consequent stabilization of HIF-1α even under conditions where it would normally be targeted for proteolysis. This stabilization may drive a transcriptional program that promotes extracellular matrix deposition and fibrosis over angiogenesis, aligning with the clinical phenotype of Gliotic PDR characterized by fibrovascular membrane formation rather than active neovascularization.

This interpretation is consistent with the established biology of the HIF pathway: *EGLN1* acts as a rheostat that adjusts HIF-1α levels in response to oxygen availability (52). Epigenetic modulation of *EGLN1* expression—whether through hypomethylation (as in adaptive responses to hypoxia) or hypermethylation (as potentially in Gliotic PDR)—can therefore shift this rheostat, tipping the balance between angiogenic and fibrotic outcomes.

However, given the exploratory nature of this analysis, the lack of multiple testing correction, and the complex, locus-specific patterns observed, these findings should be interpreted as hypothesis-generating signals requiring validation in larger cohorts and functional studies in ocular tissues. Future investigations should examine whether the differential methylation of specific *EGLN1* CpG sites correlates with gene expression levels in retinal cells and contributes to the fibrotic phenotype in PDR.

### 4.3 Differential methylation of other genes at the CpG site level

Beyond *EGLN1*, exploratory analysis identified multiple CpG sites across several other genes that showed nominal methylation differences between Florid and Gliotic PDR (**Table 5**). These included multiple sites within *SOD2* and *AKR1B1*, as well as single sites in *FLT1*, *HIF1A*, *PGF*, *SERPINF1*, and *TGFB1*.

Superoxide dismutase 2 (*SOD2*) encodes a mitochondrial antioxidant enzyme that protects against oxidative stress, a key pathogenic mechanism in DR (48). Differential methylation of multiple *SOD2* CpG sites between subtypes may reflect altered oxidative stress responses, though the functional consequences of these specific methylation changes remain unknown. *AKR1B1*, discussed earlier in the context of PDR progression, also showed differential methylation at three CpG sites between subtypes, suggesting that this gene may be subject to complex epigenetic regulation at multiple levels.

The remaining genes—*FLT1*, *HIF1A*, *PGF*, *SERPINF1*, and *TGFB1*—are all implicated in angiogenesis, hypoxia response, or fibrosis. The observation of single CpG sites with nominal differences in each of these genes raises the possibility of broader epigenetic dysregulation, but the lack of consistent patterns across multiple sites within each gene precludes firm conclusions.

Importantly, for all of these genes, the differences were observed only at the individual CpG site level, with no corresponding differences at the whole-gene or gene-segment level. Given the exploratory nature of these findings and the lack of multiple-testing correction, these observations should be interpreted with caution and validated in larger cohorts.

### 4.4 Limitations

There are some limitations in the present study. First, the sample size is relatively small, which limits statistical power and increases the risk of both false-positive and false-negative findings. Second, we acknowledge a methodological limitation in our statistical approach. The use of parametric tests (t-test, ANOVA) assumes normality of data distribution, which may not be fully met by percentage-based methylation data. Furthermore, we did not perform multiple testing correction (e.g., for the 16 target genes), meaning the reported P-values should be interpreted with caution as exploratory indicators rather than confirmatory evidence. Third, the lack of a non-diabetic healthy control group prevents distinguishing epigenetic changes specific to DR progression from those associated with diabetes or chronic hyperglycemia per se. In addition, the use of peripheral blood, while minimally invasive, may not fully capture the epigenetic landscape within the ocular microenvironment (e.g., retina, vitreous, fibrovascular membranes). This is a critical limitation when studying a localized disease like PDR. Finally, all participants had T2DM, limiting generalizability to Type 1 diabetic retinopathy. Other potential confounders, such as medications and comorbidities, were also not fully controlled for.

### 4.5 Future perspectives

Future studies are essential to validate and extend these preliminary observations. Priorities should include: 1) Conducting well-powered studies with larger, matched cohorts that apply appropriate non-parametric statistics and multiple testing corrections; 2) Investigating methylation patterns in direct ocular samples (e.g., vitreous humor, epiretinal membranes) to confirm local relevance; and 3) Performing functional assays to determine the biological consequences of the observed methylation changes. Only through such rigorous follow-up can the potential of these epigenetic signatures be fully evaluated. Furthermore, to directly address the limitation of using peripheral blood, methylation analysis of matched vitreous humor and epiretinal membrane samples from the same cohort is currently in progress. The results will be critical for validating the local relevance of the blood-based signatures identified in this study.

In summary, this exploratory study provides the first evidence that Florid and Gliotic PDR may be associated with differential methylation patterns at specific CpG sites and gene segments. While no statistically significant differences were observed at the whole-promoter level for any of the 16 target genes between the two subtypes, segmental and CpG-level variations—particularly within *EGLN1*—suggest potential epigenetic heterogeneity that may contribute to the distinct clinical phenotypes of angiogenesis-dominant (Florid) versus fibrosis-dominant (Gliotic) PDR.

Several genes showed hypomethylation in PDR patients compared to non-proliferative controls. In Florid PDR, *AKR1B1* hypomethylation may enhance polyol pathway activity and oxidative stress, while *MMP9* hypomethylation aligns with its established role in extracellular matrix degradation and neovascularization (49).

In Gliotic PDR, hypomethylation of *EPO* and *KDR* may promote fibrovascular proliferation through angiogenic and inflammatory pathways (28, 34), and *UCP1* hypomethylation—though modest in magnitude—could reflect compensatory anti-inflammatory or metabolic adaptations (41).

These preliminary findings offer a foundation for future research into subtype-specific epigenetic signatures in PDR. However, given the exploratory nature of this study, the small sample size, and the lack of multiple testing correction, all observations require rigorous validation in larger, independent cohorts and in ocular tissues. Future studies should also investigate the functional consequences of the identified methylation changes and explore their potential as minimally invasive biomarkers for risk stratification or as guides for subtype-specific therapeutic strategies.

## 5 Conclusion

In summary, this exploratory analysis identified differential methylation patterns at specific gene segments and CpG sites associated with Florid and Gliotic PDR. These hypothesis-generating findings provide a foundation for future research into epigenetic mechanisms underlying PDR heterogeneity, but rigorous validation in larger cohorts and ocular tissues is necessary before their clinical utility can be assessed.

## Data Availability

The datasets presented in this article are not readily available. Requests to access the datasets should be directed to rxt0820@163.com.
